# Development of hepatocellular carcinoma in a patient 13 years after sustained virological response to interferon against chronic hepatitis C: a case report

**DOI:** 10.1186/1757-1626-2-18

**Published:** 2009-01-07

**Authors:** Tsuyoshi Mashitani, Hitoshi Yoshiji, Masaharu Yamazaki, Yasuhide Ikenaka, Ryuichi Noguchi, Masatoshi Ishikawa, Hideto Kawaratani, Norihide Matsuo, Masahito Uemura, Junichi Yamao, Masao Fujimoto, Akira Mitoro, Masahisa Toyohara, Motoyuki Yoshida, Masayoshi Sawai, Chie Morioka, Tatsuhiro Tsujimoto, Mitsuteru Kitade, Kosuke Kaji, Yosuke Aihara, Hiroshi Fukui

**Affiliations:** 1 Third Department of Internal Medicine, Nara Medical University, Shijo-cho 840, Kashihara, Nara 634-8522, Japan

## Abstract

**Background:**

Although several recent reports have shown that hepatocellular carcinoma (HCC) developed in patients with chronic hepatitis C (CH-C) even after having a sustained virological response (SVR) to interferon (IFN) therapy, it is not common for HCC to develop more than 10 years after SVR.

**Case presentation:**

A 73-year-old Japanese man with CH-C who achieved SVR to IFN therapy 13 years ago was admitted into our hospital because of huge multiple liver tumors along with marked elevation of the tumor markers. Several diagnostic modalities strongly suggested HCC, and we performed histopathological examination. After confirming the diagnosis as well-differentiated HCC, we successfully treated these tumors with intensive combination therapies.

**Conclusion:**

Our report highlights the need for careful follow-up for more than 10 years even if the patients with CH-C achieve SVR to IFN therapy.

## Background

Hepatitis C virus (HCV) infection is the major cause of chronic liver diseases and death, not only in Japan but also throughout the world [1]. Continuous inflammation associated with HCV induces hepatic fibrosis that can lead to liver cirrhosis and hepatocellular carcinoma (HCC). The goal of therapy for chronic hepatitis C (CH-C) is eradication of HCV. Recently, anti-viral therapy against HCV has been markedly improved, especially treatment with interferon (IFN) and anti-virus agents such as ribavirin [2]. A sustained virological response (SVR), defined as seronegative polymerase chain reaction for HCV-RNA 6 months after cessation of therapy, has increased from around 5% with IFN monotherapy to almost 50% with the combination of pegylated-IFN (Peg-IFN) and ribavirin [3]. IFN therapies for chronic hepatitis C, in general, have proved effective to improve hepatic inflammation, ameliorate fibrosis and may therefore reduce the risk of HCC [4]. Furthermore, achievement of SVR after IFN therapy was reportedly associated with reduction of the liver-related mortality and the risks of complications and HCC development [5]. However, several investigators have recently disclosed that HCC did develop even after achievement of SVR to IFN in patients with CH-C [6,7]. In most of these reported cases, HCC development could be noticed within 5 years after SVR [7]. The possible existence of a small and invisible HCC, which was undetectable by various imaging procedures at the time of IFN therapy, is considered responsible for the development of HCC after complete response to IFN [8,9]. Therefore, it has been considered that HCC rarely develops long after HCV eradication. It is not common for HCC to develop more than 10 years after the achievement of SVR [9]. It is well known that the risk of HCC increases with the degree of liver fibrosis, and that HCV-related HCC rarely develops in patients without advanced fibrosis [10,11]. Similarly, among patients with SVR, advanced histological stage of disease has been linked with the development of HCC [6,7]. We report herein a rare case of a patient with CH-C that developed 13 years after SVR associated with only mild liver fibrosis.

## Case presentation

**Figure 1:**
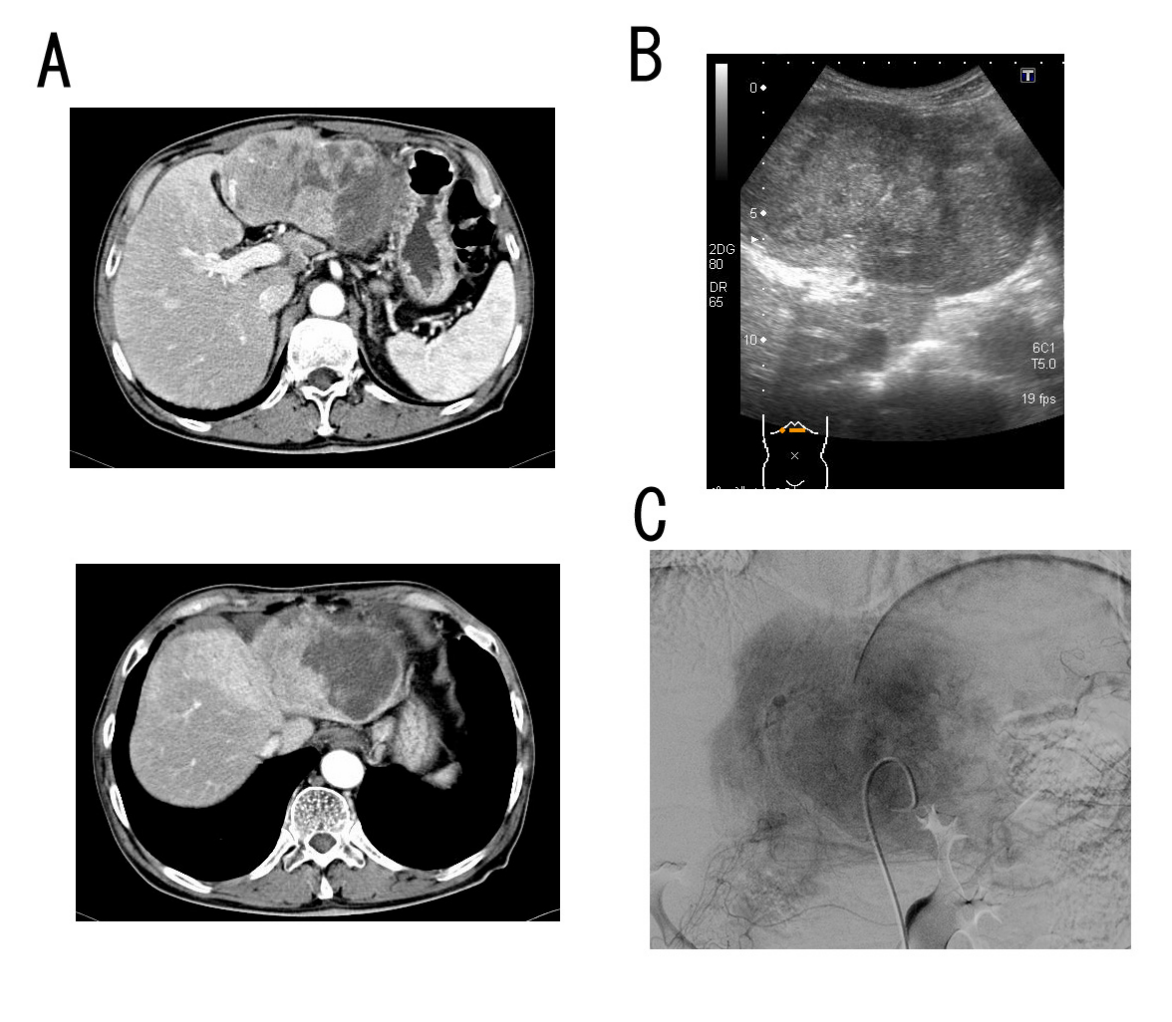
**Several imaging features of the tumors that developed in our patient 13 years after SVR**. (A): Enhanced computed tomography (CT) scans in the arterial phase at the time of admission. A huge enhanced liver tumor about 13 cm in greatest diameter in the left lobe and another tumor about 3 cm diameter in segment V could be observed. (B): An ultrasonogram (US) showing large tumors suggesting invasion of the portal vein and bile ducts. (C): Celiac angiography showing marked multiple tumors' staining in both lobes. These imaging features strongly indicate HCC.

**Figure 2:**
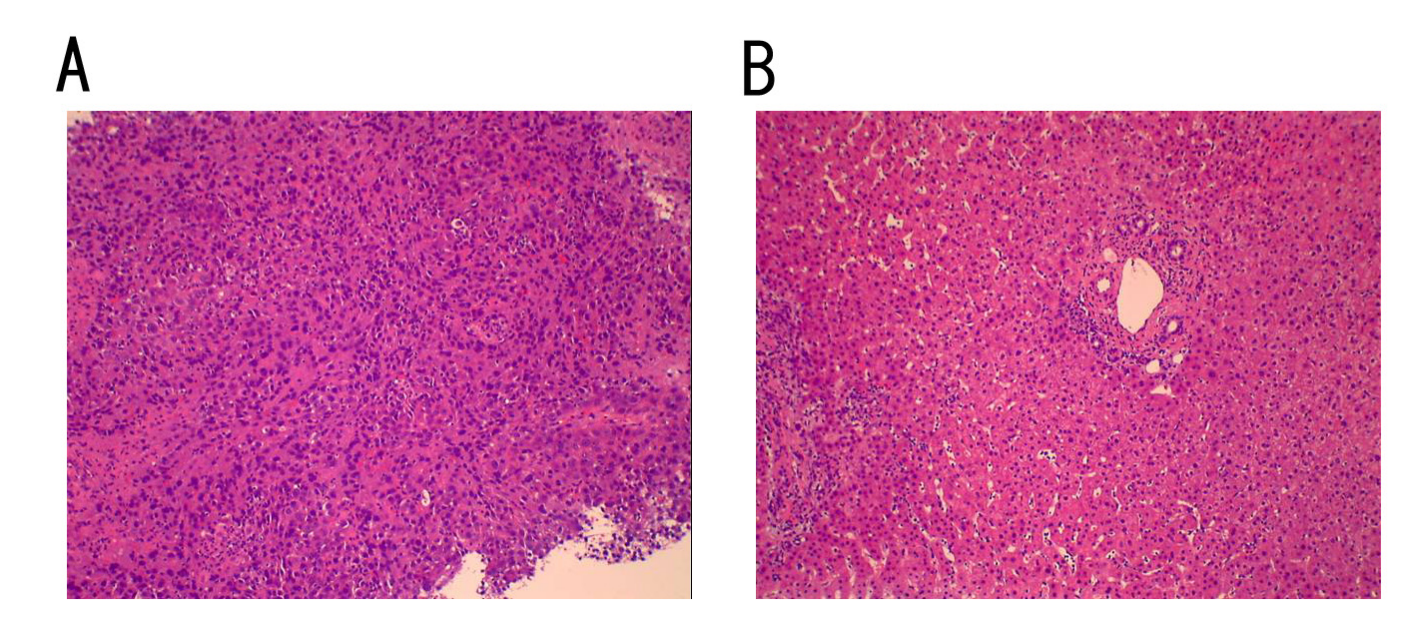
**Photomicrograph of the liver tumor**. (A):Histopathological examination revealed that the tumor lesion was well-differentiated HCC (×100). (B): The non-neoplastic adjacent lesion showed only mild fibrosis development, which was not a feature of advanced liver cirrhosis (×100).

**Figure 3:**
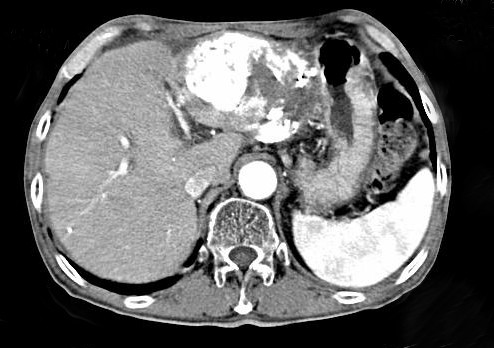
**Enhanced CT scans after the combination treatment with TAE and stereotactic radiosurgery**. The tumor size and vascularity improved, along with the significant reduction of the tumor markers (AFP and PIVKA-II). The serum level of AFP and PIVKA-II decreased from 164.9 ng/ml and 3692 mAU/ml to 14.4 ng/ml and 32 mAU/ml, respectively.

A 73-year old man was first referred to our hospital in 1992 due to liver dysfunction, and was diagnosed as having CH-C. In the following year, IFN-alpha monotherapy (3 MU 3 times a week for 24 weeks) was performed after histopathological confirmation of CH-C. With this therapy, HCV-RNA became negative and SVR was achieved. The liver functions were normalized and the histological findings of the liver also improved. The patient subsequently was followed up within HCC surveillance based on biochemical and diagnostic several modalities, such as ultrasonography (US) and computed tomography (CT), until 2000. During the follow-up period, the liver function tests were normal and HCV-RNA always tested negative on the annual testing after completion of the therapy. He willingly dropped out the follow-up since 2000. In 2006, 13 years after the achievement of SVR, he was referred to our hospital again complaining of epigastralgia. At that time, although HCV-RNA was still negative, mild liver dysfunction was noticed along with a marked increase of tumor markers of HCC, namely alpha-fetoprotein (AFP) and des-gamma-carboxy prothrombin (DCP: PIVKA-II); being 164.9 ng/ml and 3692 mAU/ml, respectively. Enhanced CT and US revealed huge liver tumors about 13 cm in greatest diameter in the left lobe invading the bile ducts and another tumor about 3 cm diameter in segment V (Figs. 1A and 1B, respectively). In the arterial phase of CT, these tumors were markedly enhanced followed by a relatively quick wash out in the equilibrium phase. Similarly, angiography revealed markedly hypervascular nodules in the same lesions, strongly indicating that these tumors were HCC (Fig. 1C). As described above, it is not common for HCC to develop more than 10 years after the achievement of SVR, and HCV-RNA still tested negative. To confirm the characteristic features of the tumor, we performed liver biopsy and confirmed that this tumor was well-differentiated HCC (Fig. 2A). Only mild fibrosis development could be observed in the adjacent non-cancerous lesions (Fig. 2B). These tumors were treated by intentional combination therapy, i.e., transcatheter arterial chemoembolization (TAE) and stereotactic radiosurgery for residual HCC and bile ducts invasion. After these therapies, the level of tumor markers significantly decreased (AFP: 14.4 ng/ml, PIVKA-II: 32 mAU/ml). CT scanning revealed a remission status of these tumors (Fig. 3), and he was discharged from our hospital to be followed up in the outpatient clinic.

## Discussion

HCC is now one of the most common malignancies in the world with an estimated annual incidence of more than one million new cases per year, and HCV infection is now one of the predominant causes of HCC [1]. The ultimate goal of therapy for CH-C is to prevent HCC and improve the long-term prognosis [5]. IFN is widely used for eradication of HCV, and IFN therapy is known to lower the incidence of HCC, especially in patients who achieved SVR [5]. However, recent studies revealed that the risk of developing HCC still exists even after SVR. Previous studies showed that HCC develops in 2.3–4.2% of patients of CH-C with SVR [6,7]. Development of HCC after IFN therapy should be considered when the interval between SVR and detection of HCC is at least a couple of years to rule out the possibility of pre-existing latent microscopic tumors. Since most tumors of HCC that develop in patients with SVR were usually detected within 5 years, several investigators speculated that HCC was already present but too small to be detected at the time of completion of IFN therapy [8,9]. This speculation is not the case in our patient, since SVR was achieved 13 years ago and no HCV-RNA could be detected when huge HCC appeared. Therefore, another possible mechanism should be considered.

Among the patients with SVR, the degree of liver fibrosis, advanced age, male sex, and alcohol intake, are higher serum alanine aminotransferase (ALT) levels are well-known risk factors for the development of HCC [6,7]. Makiyama et al. showed that, in SVR patients, the risk ratio for developing HCC was more than seven times greater in patients over 55 years of age, and was more than twice as high in patients who had advanced histological stage of the disease [6]. It has been reported that male sex is associated with a two- to three-times increased risk of HCC as compared with females [12]. Also, recent reports have suggested that obesity and diabetes mellitus were associated with an increase risk of HCC development in patients with HCV-related cirrhosis [13]. Our patient had no alcohol abuse, high ALT, or advanced fibrosis. Furthermore, the body mass index (BMI) of this patient is 21.5 and his fasting blood glucose was 85 mg/dl, both of them were within the normal ranges. Collectively, since only the age and sex were the only risk factors in our patient, it is not likely that these factors are the major reasons for huge HCC development 13 years after SVR.

Although the mechanism of HCC development in patients with SVR is still unclear, several studies have indicated that occult infection of hepatitis B virus (HBV) may be one of the carcinogenic factors [7]. It is well known that integration of the HBV genome into the host DNA induces HCC development in HBV-related liver disease [14]. Several investigators have shown that HBV DNA integration could be observed in some CH-C patients with SVR who developed HCC [15]. Although occult infection of HBV was not examined, anti-hepatitis B core antibody (HBcAb) was positive in this patient. It may be possible that occult HBV infection developed in this patient and played some role in the HCC development 13 years after SVR. Further studies are required in the future to elucidate the possible mechanism involved.

In conclusion, the risk of HCC development in patients with CH-C and SVR cannot be underestimated. Especially if the patients had at least one of the risk factors, e.g., the advanced histological fibrosis stage, advanced age, male sex, alcohol intake, higher serum ALT, obesity, diabetes mellitus, and history of HBV infection, an annual follow-up with strict surveillance program for HCC should be performed for more than 10 years after the completion of IFN therapy.

## Competing interests

The authors declare that they have no competing interests.

## Authors' contributions

TM and MK provided the clinical case, consented the patient, conceived the study, participated in its design, assisted with data collection, and coordinated and helped to draft the manuscript. HY undertook the literature and contributed to the writing and literature review. MY, YI, RN, MI, HK, KK, MU, JY, MF, AM, MT, MS, MY, CM, YA and HF were responsible for diagnosis, patient management and review. All authors read and approved the final manuscript.

## Consent

Written informed consent was obtained from the patient for publication of this case report and any accompanying images. A copy of the written consent is available for review by the Editor-in-Chief of this journal.
